# Explaining Accessibility and Satisfaction Related to Healthcare: A Mixed-Methods Approach

**DOI:** 10.1007/s11205-016-1371-9

**Published:** 2016-06-02

**Authors:** Pablo Cabrera-Barona, Thomas Blaschke, Stefan Kienberger

**Affiliations:** Interfaculty Department of Geoinformatics - Z_GIS, University of Salzburg, Schillerstraße 30, 5020 Salzburg, Austria

**Keywords:** Healthcare, Accessibility, Satisfaction, Composite index

## Abstract

Accessibility and satisfaction related to healthcare services are conceived as multidimensional concepts. These concepts can be studied using objective and subjective measures. In this study, we created two indices: a composite healthcare accessibility index (CHCA) and a composite healthcare satisfaction index (CHCS). To calculate the CHCA index we used three indicators based on three components of multidimensional healthcare accessibility: availability, acceptability and accessibility. In the indicator based on the component of accessibility, we included an innovative perceived time-decay parameter. The three indicators of the CHCA index were weighted through the application of a principal components analysis. To calculate the CHCS index, we used three indicators: the waiting time after the patient arrives at the healthcare service, the quality of the healthcare, and the healthcare service supply. These three indicators making up the CHCA index were weighted by applying an analytical hierarchy process. Three kinds of regressions were subsequently applied in order to explain the CHCA and CHCS indices: namely the Linear Least Squares, Ordinal Logistic, and Random Forests regressions. In these regressions, we used different independent social and health-related variables. These variables represented the predisposing, enabling, and need factors of people´s behaviors related to healthcare. All the calculations were applied to a study area: the city of Quito, Ecuador. Results showed that there are health-related inequalities in regard to healthcare accessibility and healthcare satisfaction in our study area. We also identified specific social factors that explained the indices developed. The present work is a mixed-methods approach to evaluate multidimensional healthcare accessibility and healthcare satisfaction, incorporating a pluralistic perspective, as well as a multidisciplinary framework. The results obtained can also be considered as tools for healthcare and urban planners, for more integrative social analyses that can improve the quality of life in urban residents.

## Introduction

Accessibility is a widely studied analytical topic that supports the understanding of people´s access to social services, which can ensure or improve their quality of life (Kwan 2013). Health is a multi-faceted concept in which the analysis of healthcare services is an important issue that necessitates multivariate approaches (Klomp and de Haan 2010). Consequently, accessibility to healthcare services is an important subject to be considered in the study of healthcare, due to the fact that access to healthcare can be thought of as a facilitator of overall population health (Guagliardo 2004). Yet, accessibility to healthcare services is a multidimensional and complex concept (Andersen 1995) not only limited to distance measures, but also to subjective measures (Comber et al. 2011). In addition, problems of accessibility create unmet healthcare needs, which may result not only from distance barriers, but could also be the result of an unavailability of healthcare services and individual acceptability of these services (Aday and Andersen 1974; Chen and Hou 2002).

Multidimensional healthcare accessibility is explained by different factors, e.g. the factors of Andersen´s behavioral model: predisposing, enabling and need factors (Aday and Andersen 1974; Andersen 1995; Arcury et al. 2005; Cavalieri 2013). This multidimensional accessibility can also be classified in the dimensions of availability, accessibility and acceptability (Cavalieri 2013; Chen and Hou 2002; Penchansky and Thomas 1981). There are another two additional dimensions, i.e. accommodation and affordability (Penchansky and Thomas 1981). However, accommodation includes healthcare service features that can be incorporated into a measure of accessibility (Rosero-Bixby 2004), and affordability includes concepts that can be represented by the factors of Andersen´s behavioral model (Aday and Andersen 1974; Arcury et al. 2005).

The accessibility dimension refers to the separation between the population and the healthcare services (Delamater 2013), e.g., the travel time from a population’s location to the healthcare service’s location. Availability usually refers to the number of healthcare services that a patient can choose from (Penchansky and Thomas 1981), but can also consider other indicators, such as the appointment waiting time (Aday and Andersen 1974; Cavalieri 2013). Appointment waiting time is the time a person has to wait to get an appointment (waiting list time). The combination of the two dimensions ‘accessibility’ and ‘availability’ is also referred as “spatial accessibility” (Guagliardo 2004); therefore, travel time and the time to get an appointment can be considered as measures that explain “spatial accessibility”. Acceptability comprises the patient´s attitudes, concerns and beliefs (Cavalieri 2013; Penchansky and Thomas 1981).

Accessibility is also related to financial barriers and transportation problems (Cavalieri 2013). However, these variables can also be considered as factors of the health behavioral model (Aday and Andersen 1974; Arcury et al. 2005). Accessibility can also be explained by geographic variables that refer to the “friction of space” (Kwan 1998; Lin et al. 2002), linking the location of healthcare services and the location of patients (Guagliardo 2004). Previous work on healthcare accessibility has focused on using location-based approaches that basically consider gravity models of accessibility (Apparicio et al. 2008; Hu et al. 2013; Fransen et al. 2015). These measures of accessibility require the calculation of distance-decay parameters (Kwan 1998), which are generally difficult to calculate because they demand analyses of very detailed empirical data (Hu et al. 2013; Luo and Qi 2009; Luo and Whippo 2012). The values of these kinds of parameters vary by the kind of service (Gesler and Cromartie 1985), population characteristics (Bronstein and Morissey 1990), and people´s mobility (Arcury et al. 2005). Mobility can be explained by the estimated flow of people between geographical locations (Dennet 2012) under consideration of spatial interaction models (Desta 1990).

Using time as a proxy for geographical distances is a useful option to assess accessibility (Kwan 1998). In our study we expand the traditional geographical analyses of accessibility by incorporating perceived travel time to the healthcare services. We thereby developed a qualitative–quantitative approach, taking into consideration the importance of using individual healthcare perceptions in healthcare accessibility analyses (Hawthorne and Kwan 2012).

The satisfaction of the healthcare service users is a health outcome that can complement the study of healthcare accessibility. This kind of satisfaction comprises patients´ judgments of the quality of healthcare they received (Aday and Andersen 1974). Satisfaction is an important component of quality of life that depends on the subjective feelings of individuals, which are the product of individual interaction with objective life conditions (Felce and Perry 1995). In the case of healthcare analyses, the satisfaction of patients can be considered as an evaluation of a received healthcare service (Pascoe 1983). There is no general consensus regarding the concept of patient satisfaction (Avis et al. 1997; Hekkert et al. 2009; Jenkinson et al. 2002; Pascoe 1983), therefore it is necessary to be aware of the kind of satisfaction that is being evaluated.

Healthcare satisfaction is proven to be a function of patient–physician interaction, where the quality of the service is proportional to a patient´s satisfaction (Hawthorne and Kwan 2012; Pascoe 1983). The quality of healthcare influences the perceived satisfaction of a patient (Hekkert et al. 2009). Other variables have been related to healthcare satisfaction, such us the physical characteristics and the resources of the healthcare service (Rosero-Bixby 2004; Sitzia and Wood 1997). The patient´s satisfaction may not only be explained by the patient´s experience at the healthcare service, but also by the health system in general or by the characteristics of this service (Bleich et al. 2009; Hekkert et al. 2009). These characteristics can be represented by the kind of healthcare service (Rosero-Bixby 2004), e.g., specialized hospitals, general hospitals or healthcare centers. On the other hand, the waiting time once the patient arrives at the healthcare service has also been considered as a key variable influencing a patient´s satisfaction (Aday et al. 1980; Hawthorne and Kwan 2012; Pascoe 1983; Sitzia and Wood 1997).

Measures of satisfaction and accessibility can be considered as dependent variables of predisposing, enabling and need factors (Aday and Andersen 1974; Andersen 1978, 1995; Andersen and Newman 2005; Cavalieri 2013; Pascoe 1983). These factors represent population characteristics (Aday and Andersen 1974), and are also defined as individual determinants of the use of healthcare services (Andersen and Newman 2005). The use of healthcare services is associated to healthcare accessibility, and is directly related to the satisfaction of the patients (Andersen 1995). Predisposing factors include demographic and social variables, such as age, gender, marital status, and education (Andersen and Newman 2005). Common variables studied as enabling factors are income and health insurance (Andersen and Newman 2005; Arcury et al. 2005). Specifically, health insurance is an important determinant of healthcare seeking behavior (Frank and Lamiraud 2009; Schram and Sonnemans 2011; van den Berg et al. 2008). Need factors are related to the individual illness level, which can be represented by the perceived health status of a person (Aday and Andersen 1974; Andersen 1995).

A key issue arises from the described background: there has been little discussion about the development of mixed methods that consider subjective and objective measures to calculate composite indices of accessibility and satisfaction related to healthcare services. So far, there has only been a limited analysis of using different statistical approaches to evaluate measures of healthcare accessibility and satisfaction in relation to predictors such as the factors of the health behavioral model.

The purpose of our study is to develop a composite healthcare accessibility (CHCA) index and a composite healthcare satisfaction (CHCS) index using objective and subjective indicators, applying mixed-methods approaches. Then, using predisposing, enabling and need factors as predictors, we aim to explain these indices by evaluating their response to these factors. We argue that particularly the combination of subjective and objective measures can support the construction of indices, which may likewise support a better understanding of healthcare disparities. We also argue that the indices developed in this study can be explained by different social factors related to healthcare-seeking behavior.

## Materials and Methods

### Survey Design and Participants

**Fig. 1:**
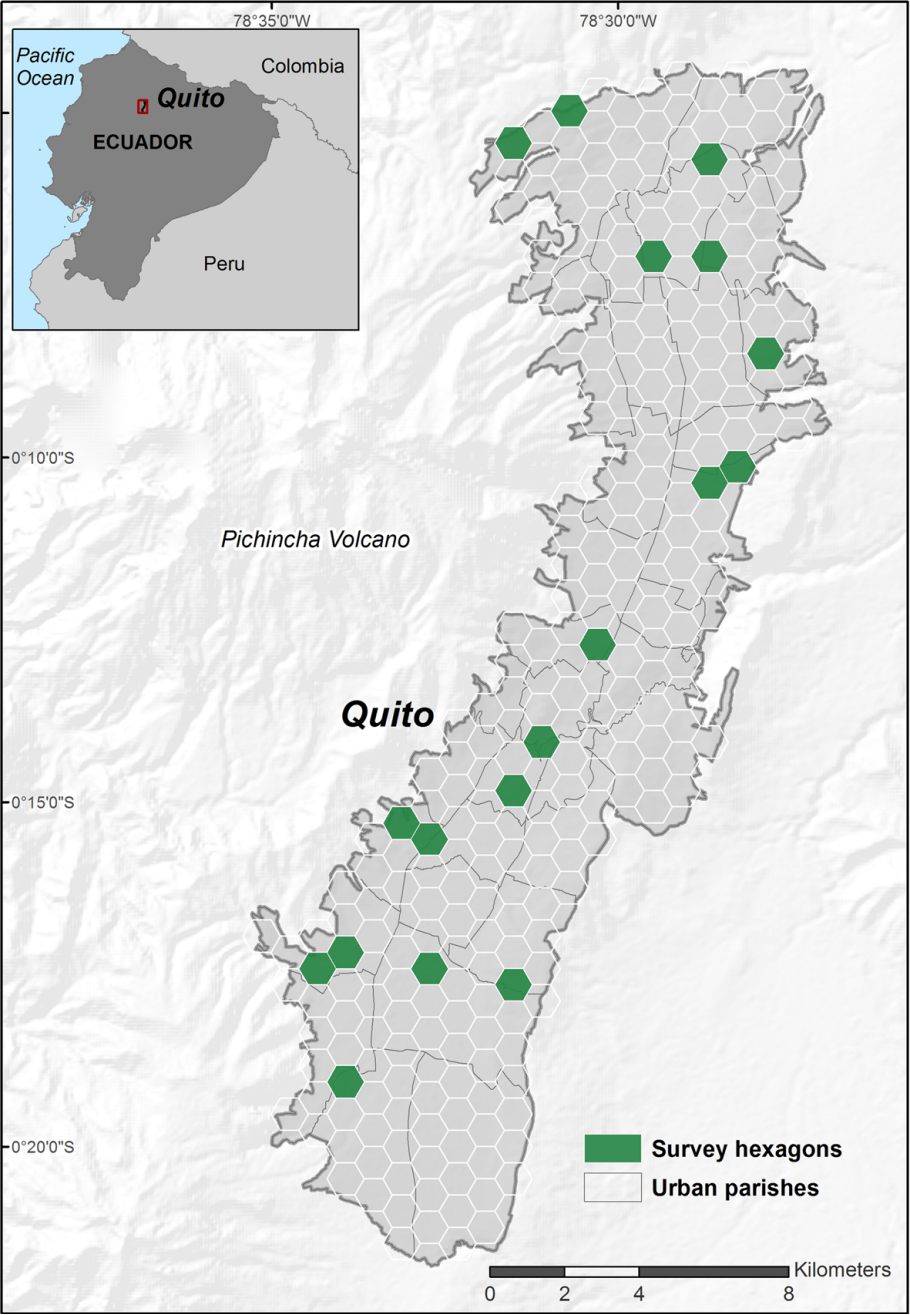
Study area

A survey was carried out during 5 weeks in the months of July, August and October 2014 in Quito, the capital city of Ecuador (Fig. 1). A two-stage sampling strategy was carried out. The first stage was the creation and selection of sampling clusters. At this stage, the land use/land cover map of Quito was first visualized in a geographic information system (GIS), and then residential areas were extracted. Thereupon, the study area was divided into one kilometer wide (diameter) hexagons. The reason for choosing hexagons is that their tessellation leads to very efficient sampling, since this kind of polygon are the most compact and regular polygons that can form a continuous grid. The one-kilometer width for each sampling hexagon was chosen since this was large enough to find sufficient persons to interview, while not being too large and thus causing logistic barriers for interviewers. The study area was divided into hexagons (296 hexagons covered the study area) using a GIS tool, and then 18 hexagons were randomly chosen. This number of hexagons was selected considering the interviewers´ capacity in terms of time, and their financial resources for travelling within the city. In the second stage of our sample strategy, pseudo-random interviews were carried out in the field within each sampling hexagon. The numbers of interviewees in each sampling hexagon varied as a function of the population density inside the hexagon. Interviewers covered each hexagon by carrying out door-to-door interviews in households where people were willing to participate. The obtained response rate was 61 %, which means that more than half of the people that were approached accepted the interview. A total of 471 valid questionnaires were thereby obtained for this study. The margin of sampling error was ±4, with a level of confidence of 95 %.

An online questionnaire was also carried out in April 2015 to obtain experts´ judgments, to subsequently implement the analytical hierarchy process (AHP), a method that is explained in Sect. 2.2.2. We obtained 38 experts´ judgments. The experts are professionals and decision makers that work in the fields of social sciences and environmental sciences, e.g., health, geography and sociology.

### Procedure

The conceptual framework summarized in the introduction section was used to select indicators to subsequently construct two indices: a composite healthcare accessibility index (CHCA) and a composite healthcare satisfaction index (CHCS).

The information to construct and explain the CHCA and CHCA indices was obtained in the survey carried out in the study area. The survey comprised in-depth interviews administered through questionnaires that contained Likert scale questions, multiple choices questions and open questions. The questions for the interviewees were related to healthcare accessibility and the quality of the healthcare received, as well as to socio-economic and demographic parameters.

In the case of the CHCA index, a perceived time-decay parameter was calculated first. This parameter was incorporated in the calculation of an indicator of accessibility. This indicator and two more indicators, the acceptability and availability indicators, where used to construct the CHCA index. Indicators of waiting time, quality of healthcare and healthcare service supply were used to construct the CHCS index. Predisposing, enabling, and need factors were utilized as independent variables in order to explain the CHCS and the CHCA indices by using three different kinds of regressions: Linear Least Squares, Ordinal Logistic and Random Forests regressions.

**Fig. 2:**
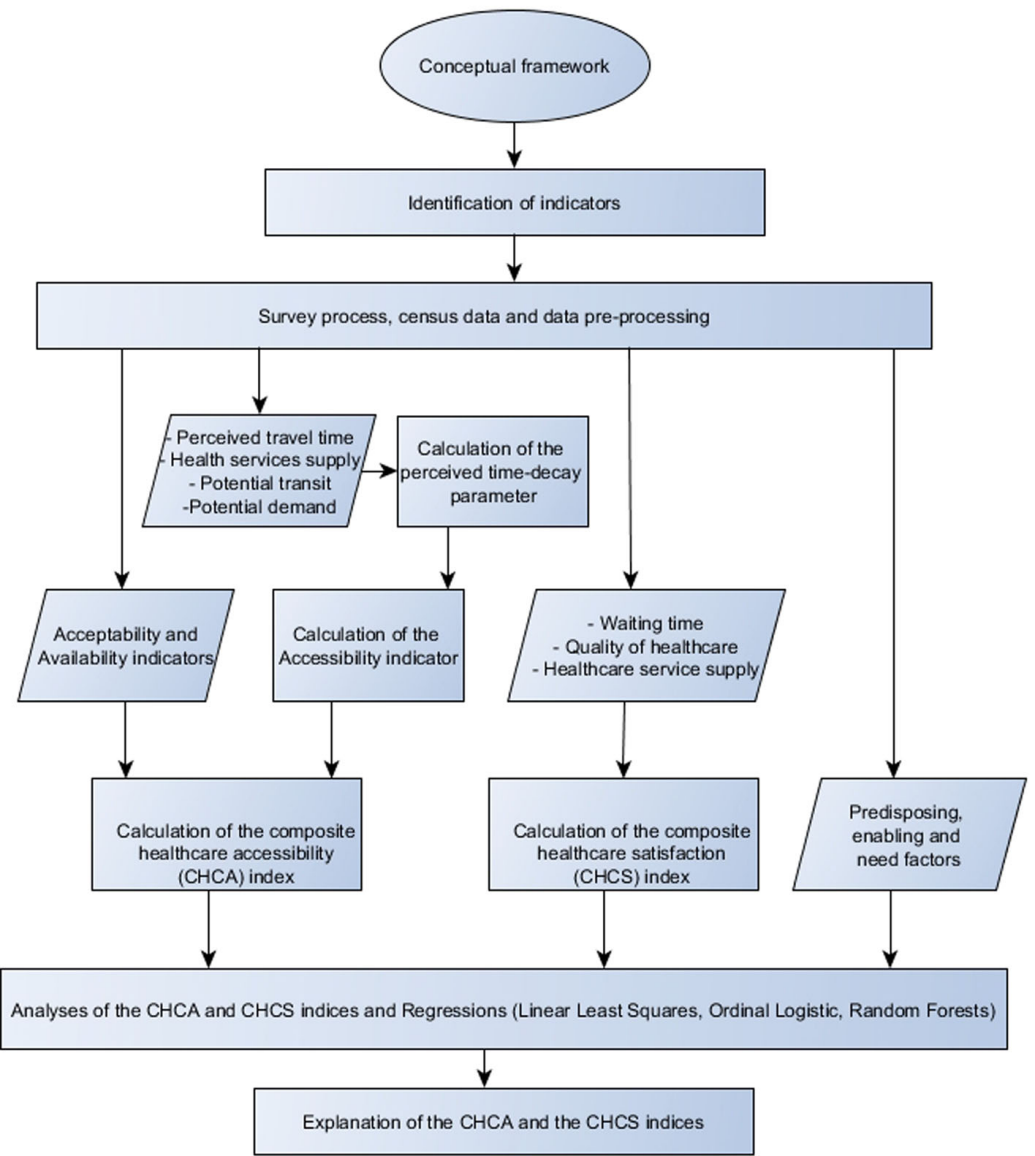
Methods workflow

An overview of the methods applied in this study is displayed in Fig. 2. These methods are elaborated in the next sub-sections.

#### Development of the Composite Healthcare Accessibility Index (CHCA)

Three different indicators composed the CHCA index: accessibility (*Acc*), availability (*Avai*) and acceptability (*Accep*). In this section, we first explain how to calculate the *Acc* indicator and its perceived time-decay parameter. Second, we explain the variables that are used to represent the *Avai* and the *Accep* indicators. Third, we indicate the calculation of the CHCA index.

The accessibility (*Acc*) can be expressed as a function of the sum of the “economic activities” *E*_*j*_, the distance to the service´s location, and a distance-decay parameter called *β* (Caschili et al. 2015):

$$Acc = E_{i} f(\beta ,d_{ij} )$$

Using an exponential form to express the function of *β* and *d*_*ij*_ and considering a time measure instead of a distance measure, we can formulate the following equation (Kwan 1998; Caschili et al. 2015):

$$Acc = E_{i} e^{{ - t_{ij\beta } }}$$

For our study, *E*_*j*_ represents the sum of healthcare services accessible in a place *i*. We chose the smallest politic-administrative areas in Ecuador, namely Parishes, to define the place *i*. *t*_*ij*_ represents the time that an interviewee (healthcare user) reported to have taken to travel to the healthcare service. *β* is the perceived time-decay parameter. This parameter is based on spatial interaction and is explained down below.

Some metrics of accessibility are based on spatial interaction models, which are based on the potential number of individuals that can travel to reach certain destinations (Caschili et al. 2015). In the field of healthcare, this potential number of individuals is also known as potential demand (Andersen 1995; Guagliardo 2004; Luo and Qi 2009). Potential demand exists when a population coexists in space and time with an able healthcare service (Guagliardo 2004). A distance decay parameter can be obtained by linking spatial interaction data to gravity-type regressions and models (Crooks and Schuurman 2012; Johnston 1973; Kwan 1998; Luo and Whippo 2012; Mikkonen and Luoma 1999).

Considering a geographical gravity model derived from Newton´s law of gravity, which represents an interaction between place *i* and *j* (Liu et al. 2014):

$$I_{ij} = \frac{{kP_{i} P_{j} }}{{f\left( {d_{ij} } \right)}}$$

where *I*_*ij*_ represents the interaction *i*–*j*, *P*_*i*_ is the propulsivneness of place *i*, *P*_*j*_ is the attraction of place *j* and *d*_*ij*_ is the distance between place *i* and *j*. A function *f*(*P*_*i*_ *P*_*j*_) can be equivalent to a function *f*(*D*, *S*), where *D* represent a potential demand for a service (propulsivneness) and *S* represent the service supply (attractiveness). A modified gravity model (Crooks and Schuurman 2012) for one specific place *i* to one specific health service *j* can be expressed as:

$$A_{i} = \frac{{S_{j} }}{{D_{j} f\left( {t_{ij} } \right)}}$$

where *A*_*i*_ is the access measure, *S*_*j*_ represents a measure of the healthcare service supply, *D*_*j*_ is the potential demand and *t*_*j*_ is the travel time from one position in space to the healthcare service location. If the accessibility is defined as an interaction *I*_*ij*_ that can represent the potential transit of people from one area *i* to a service area *j* (Caschili et al. 2015), we can say that this transit is a function of the demand (“propulsiveness”), healthcare service supply (“attractiveness”) and the travel time or distance between *i* and *j* (Desta 1990). Therefore, *I*_*ij*_ results in an equivalent measure of *A*_*i*_. Considering this approach, we can formulate a new spatial interaction-gravity model formula for a specific healthcare user located in *i* that travels to a healthcare service located in *j*:

$$I_{ij} = k\frac{{S_{j}^{\gamma } }}{{D_{j}^{\alpha } t_{ij}^{\beta } }}$$

where *I*_*ij*_ is the potential transit represented by the population living in the same administrative area (in this study, a Parish) *i* where the healthcare user lives; *k* is a constant.

*S*_*j*_^*γ*^ is the supply measure of the healthcare service that can be represented by the kind of service: health center (includes particular physician), basic hospital, or specialized hospital. A 1–3 score was used to value this variable, in which 1 represents the health center, 2 represents the basic hospital, and 3 represents the healthcare service with the greatest variety of services, the specialized hospital.

*D* ^*α*^ is the potential total demand including the population represented by *I*_*ij*_, and the population living in the same administrative area *j* where the healthcare service is located. Populations of Parishes were extracted from the 2010 Ecuadorian Population and Housing Census.

Finally, *t*_*ij*_^*β*^ represents the time that an interviewee (healthcare user) reported to have taken to travel to the healthcare service.

By applying logarithms in the last equation, a new equation is obtained:

$$lnI_{ij} = lnk + \gamma lnS_{j} - \propto lnD_{j} - \beta lnt_{ij}$$

And calculating an ordinary least squares regression the value of the parameter *β* (perceived time-decay parameter) was estimated: 0.024. This parameter is included in the equation of *Acc* to obtain the indicator of accessibility for the CHCA index.

Thereupon, it is shown the definition of the variables used to represent the indicators of availability (*Avai*) and acceptability (*Accep*). These variables were extracted from the survey questionnaire.

The appointment waiting time (in hours) to receive healthcare was used to represent *Avai*. A variable defined as the main reason the patient chose a healthcare service was used to represent *Accep*. If the patient decided to go to the healthcare service because he or she had confidence in the physician/healthcare service, this indicator receives a score of 3. If the patient decided to go to the healthcare service because another person recommended it, the indicator receives a score of 2. If the patient decided to go to the healthcare service because he or she has health insurance for that service, or because the service was located close to his or her home or work, the indicator is given the value of 1. For any other reasons, the value of the indicator is 0.

Once the three indicators for the CHCA index (*Acc*, *Avai*, *Accep*) were obtained, a principal components analysis (PCA) was applied to extract the weights (*w*_*Acc*_, *w*_*Avai*_, *w*_*Accep*_) of these indicators. To perform the PCA, first, the z values of *Acc*, *Avai* and *Accep* were calculated. Then, a PCA was applied, using an eigenvalue threshold of 1 and a varimax rotation. The Bartlett´s tests of sphericity yielded a significance value lower than 0.05, which confirmed the application of PCA to the chosen indicators. Two components with eigenvalues larger than 1 were obtained. The indicators´ weights were calculated using the squared factor loading matrix scaled to unity sum and the component proportion of the explained variance in the data (OECD 2008). Table 1 shows the weights extracted using PCA.

**Table 1:** Weights extracted using PCA

Indicator	Weights (w_j_)
Acc	0.45
Avai	0.29
Accep	0.26

The three indicators (*Acc*, *Avai*, *Accep*) were normalized by applying linear min–max normalization (OECD 2008), and then the *CHCA* index was calculated by adding the weighted normalized indicators:

$$CHCA = w_{Acc} \left( {Acc} \right) + w_{Accep} \left( {Accep} \right) - w_{Avai} \left( {Avai} \right)$$

#### Development of a Composite Healthcare Satisfaction Index (CHCS)

Three indicators composed the CHCI index: the waiting time (in hours) to receive healthcare attention after arriving at the healthcare service (*T*), the quality of healthcare attention received from the physician or healthcare professional (*Q*), and the healthcare service supply (*S*). The *Q* indicator is based on the last healthcare experience that an interviewee had: a 1–5 score was used, where 5 means the best quality of healthcare attention. The *S* indicator represents the latent satisfaction or utility of a healthcare service and can be considered equivalent to the *S*_*j*_^*γ*^ variable used for the calculation of the perceived time-decay parameter.

Satisfaction measures are variables related to perceptions and peoples´ feelings, and can represent complex situations in which it could be difficult for them to make or construct consistent decisions or criteria (Redelmeier and Shafir 1995). When dealing with healthcare, it is important to consider differences in patients´ preferences or criteria (Montgomery and Fahey 2001). The CHCS index indicators *T*, *Q* and *S*, can be considered as criteria where people´s judgments define the importance of each one. We use the analytical hierarchy process (AHP) to create weights for the indicators *T*, *Q* and *S*. The AHP is a multi-criteria evaluation method that works with priorities in a hierarchical structure (Saaty 1980, 1987). This process is a widely used multi-criteria method (Ho 2008) that offers a straightforward way of obtaining weights for criteria or indicators (Feizizadeh and Blaschke 2011).

AHP works with a pairwise comparison matrix to evaluate the different indicators used (Boroushaki and Malczewski 2008) by comparing the importance of each indicator with respect to the other indicators. The importance of each indicator is defined by experts´ judgments using a 1–9 scale, where, for example, 1 means *indicator Q has equal importance as indicator S,* and 9 means *indicator Q is extremely more important than indicator S* (Dolan 2008). The other odd values on the scale (3, 5, 7) can be interpreted in the following way: 3 as “moderately more important than”, 5 as “strongly more important than” and 7 as “very strongly more important than”. The even values on the scale, 2, 4, 6 and 8, are considered intermediate values between two of the values given before. For example, 2 means: “nearly equally to moderately more important than”.

To define the levels of importance for indicators *T*, *Q* and *S*, 38 experts´ judgments were obtained via the online questionnaire introduced in the Sect. 2.1 of the article. The pairwise comparison matrix was constructed using this information.

In the AHP, the pairwise comparison matrix has to be normalized (Gómez and Barredo 2005) by calculating the ratio of each value of the pairwise comparison matrix and the values sum of each column of this matrix. To obtain the weights, an eigenvalues matrix has to be calculated: the values of each row are added, and then the sum is divided by the number of the indicators used (Cabrera-Barona et al. 2015). The eigenvalues are the equivalent of the indicators´ weights.

Table 2 shows the values used to construct the pairwise comparison matrix and the indicators weights obtained.

**Table 2:** Pairwise comparison matrix

Indicator	T	Q	S	Weights
T	1			0.31
Q	2	1		0.49
S	1/2	1/2	1	0.20

Because the comparisons are obtained from subjective perceptions of the experts´ judgments, some degree of inconsistency may occur (Ho 2008). To evaluate whether there is inconsistency in the experts´ judgments, a consistency ratio (CR) can be used (Saaty 1987). The CR is the ratio between a consistency index (CI) and a random index (RI):

$$CR = \frac{CI}{RI}$$

The CI is a function of the number of indicators and the maximum eigenvector that can be obtained from the eigenvalues matrix and the pairwise comparison matrix (Gómez and Barredo 2005):

$$CI = \frac{{\lambda_{max} - n}}{n - 1}$$

In the CI equation, *n* represents the number of indicators. *λ*_*max*_ is obtained by calculating two vectors: (i) a vector that is the product of multiplying the eigenvalues matrix and the pairwise comparison matrix, and (ii) a vector obtained by dividing the values of the previous vector (i) by the values of the eigenvalues matrix. *λ*_*max*_ is the average of all the components of this final vector (Gómez and Barredo 2005).

The RI is generated from a random pairwise comparison matrix (Boroushaki, and Malczewski 2008). The value that this index can take depends on the number of indicators used. In this case, because we have three indicators, the RI value is 0.58 (Cabrera-Barona et al. 2015; Gómez and Barredo 2005).

If the CR is less than 0.10, it means that there is a reasonable level of consistency in the pairwise comparison (Boroushaki and Malczewski 2008). The CR we obtained was 0.05, which allowed us to use the AHP weights for the CHCS index.

The three indicators (*T*, *Q*, *S*) were normalized by applying linear min–max normalization (OECD 2008), and then the *CHCS* index was calculated by adding the weighted normalized indicators:

$$CHCS = w_{Q} \left( Q \right) + w_{S} \left( S \right) - w_{T} \left( T \right)$$

#### Explaining the *CHCA* and *CHCS* Indices Using Predisposing, Enabling and Need Factors

We used predisposing, enabling and need factors to explain the CHCA and CHCS indices. Different social and health-related variables were considered to represent these factors (Cavalieri 2013; Andersen 1995; Arcury et al. 2005). All the variables were extracted from the survey obtained in the field work.

For the predisposing factor, we used the following variables: gender (female/male), age, education (having tertiary education/not having tertiary education), marital status (married/not married), and employment situation (employed/unemployed). For the enabling factor, we used the variable of health insurance tenure (having health insurance/not having health insurance). For the need factor, we used the variable of self-perceived health status (poor health/good health).

Considering the CHCA and CHCS indices as dependent variables and the factors above named as independent variables, we used three kinds of regressions: Linear Least Squares (LLS) regression, Ordinal Logistic (OL) regression, and Random Forests (RF) regression. In the case of LLS regression for the CHCS index, this dependent variable had to be transformed using a Box-Cox transformation (logarithmic), in order to stabilize the residuals´ variances. In the case of the OL and RF regressions, the values of the CHCA and CHCS indices were transformed to categorical ordinal values. To do this, the two indices were normalized to values between 0 and 1. Then, the following categorical values were assigned: 1 for indices´ values lower than 0.30, 2 for values larger than 0.30 and lower than 0.6, and 3 for values larger than 0.6. For both CHCA and CHCS indices, values of 1, 2, and 3 mean low, middle and high healthcare accessibility and healthcare satisfaction, respectively.

Random Forests is a classification and regression trees technique (Breiman 2001) that uses boot-strapped regression trees that are grown with a randomized subset of predictors (Prasad et al. 2006). The predictions for a tree are based on the out-of-bag (OBB) data, which is data that corresponds to approximately one-third of the observations (Pang et al. 2006). One of the advantages of using Random Forests is that the importance of predictors can be estimated in order to better understand what happens in linear models (Grömping 2009). We used 500 trees and 2 variables splitting at each tree node.

## Results

Table 3 shows the descriptive statistics of the CHCA and CHCS indices. Most of CHCA index values (83 %) are values lower than 0.30. The mean value of CHCA is 0.22 and its minimum and maximum values are −0.08 and 0.71, respectively. The average value of the CHCS index is 0.42, and it has a minimum value of −0.01 (negative satisfaction) and a maximum value of 0.69.

**Table 3:** Descriptive statistics of CHCA and CHCS indices

	Min	Max	Mean	SD
CHCA index	−0.08	0.71	0.22	0.14
CHCS index	−0.01	0.69	0.42	0.12

**Fig. 3:**
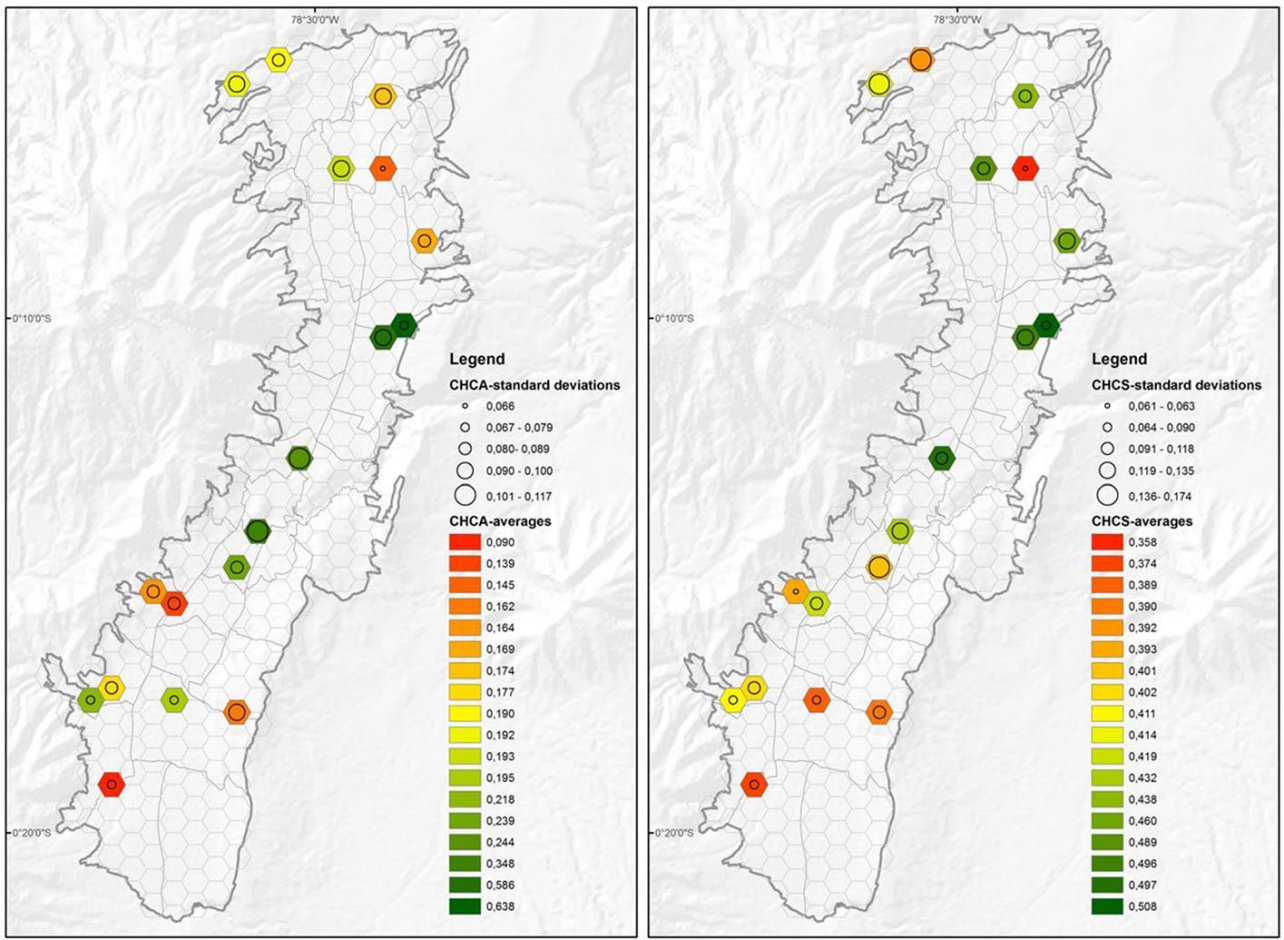
CHCA and CHCS average values in survey zones

Figure 3 shows a spatial representation of the CHCA and CHCS indices using averages and standard deviations of these indices in the survey zones.

In general, high values of CHCA and CHCS were found in the central and central-northern areas of the city. These areas are characterized by a high urban density with high levels of public services, including healthcare services. Medium and low values of CHCA and CHCS were found in southern areas, as well as in the extreme north-west of the city.

With the CHCA index, areas located in the center of the city are areas that have high mean values of CHCA, but also have high standard deviations. This means that composite accessibility varies more in the center of the city than in other areas of Quito. The area with the lowest CHCA index value (average of 0.090) is located in the south of Quito. The standard deviation in this zone (0.080) shows that there are similar low values of healthcare accessibility in this zone.

Using the CHCS index, two areas located in the north-west of the city were shown to have low mean values of CHCS, but high standard deviations, which indicate that individuals do not have very similar healthcare satisfaction characteristics in these areas. We also identified an area located in the north of the city that has the lowest values of the CHCS index, with an average of 0.358 and a very low standard deviation (0.061).

Table 4 depicts the results of the LLS regression. The factors ***education,*** ***marital status*** and ***insurance*** were found to be significant when explaining the CHCA index, while the factors ***insurance*** and ***need*** were found to be significant in explaining the CHCS index. The tenure of health insurance (significances of 0.02 for the CHCA index and 0.04 for the CHCS index) had a positive association with both indices. These results suggest that having insurance positively influence healthcare accessibility and healthcare satisfaction.

**Table 4:** Results of LLS regressions

	CHCA index		CHCS index	
	Coefficients	Significance (*p* value)	Coefficients	Significance (*p* value)
Gender	0.76	0.47	0.01	0.82
Age	0.02	0.59	0.00	0.89
Education	2.60	**0.03**	0.06	0.21
Marital status	−2.84	**0.01**	0.03	0.47
Employment	−1.04	0.38	0.00	0.98
Insurance	2.57	**0.02**	0.09	**0.04**
Need	−0.99	0.48	0.16	**0.00**

Significant values are interpreted in bold

Coefficients are unstandardized coefficients. Level of significance is at 95 % of confidence

We observed a statistically very significant association (*p* < 0.001) between the need factor and the CHCS index. For our study, this factor is represented by the self-perceived health status, considering having good health as a reference variable. Therefore, our results suggested that the self-perceived health status can be considered as a predictor of healthcare satisfaction and that having good health can slightly improve healthcare satisfaction.

Marital status (to be married) is a significant factor (significance of 0.01) for the CHCA index, and is negatively related to this index. Having tertiary education significantly and positively influences access to healthcare services (significance of 0.03).

Table 5 shows the OL regression results: ***insurance*** and ***education*** are significant factors for the two indices CHCA and CHCS. The odds ratios suggest that increasing health insurance tenure positively impacts healthcare access and healthcare satisfaction. For example, in the case of the CHCA index, the relative odds of experiencing a positive accessibility to healthcare are 1.53 times greater for respondents with health insurance than for those who do not have health insurance. In the case of the CHCS index, the relative odds of experiencing a positive satisfaction related to healthcare are 2.15 times greater for respondents with health insurance than for those who do not have health insurance. A similar interpretation can be applied for the education factor.

**Table 5:** Results of OL regressions

	CHCA index			CHCS index		
	Odds ratio	2.5 %	97.5 %	Odds ratio	2.5 %	97.5 %
Gender	1.14	0.79	1.63	1.08	0.75	1.59
Age	0.99	0.99	1.01	0.99	0.98	1.01
Education	1.79	**1.20**	**2.69**	1.65	**1.08**	**2.53**
Marital status	0.63	0.43	0.93	0.79	0.53	1.19
Employment	0.81	0.54	1.20	0.84	0.55	1.29
Insurance	1.53	**1.05**	**2.22**	2.15	**1.46**	**3.19**
Need	0.79	0.49	1.29	1.07	0.65	1.79

Significant values are interpreted in bold

Limits of 2.5 and 97.5 % are the confident intervals. These intervals represent significance at 95 % of confidence

Results of the RF regressions (Table 6) confirm the results obtained in the LLS and OL regressions: ***insurance*** and ***education*** were found to be very important factors when explaining our indices. However, when using RF, the ***age*** factor obtained the highest relevance. Nonetheless, RF results validate the important role of having health insurance for explaining our indices. The ***marital status*** and **need** factors were found to be relatively important for the indices CHCA and CHCS respectively, coinciding with the results obtained from LLS regression.

**Table 6:** Results of RF regressions

Importance of factors for CHCA index	Importance of factors for CHCS index
**Age**	**Age**
**Education**	**Insurance**
**Insurance**	**Education**
**Marital status**	**Need**
Need	Marital status
Employment	Employment
Gender	Gender

The factors in the columns are ordered according to their importance in explaining the indices. We consider the first four (bold letters) to be the most important factors

In general, having health insurance was found to be a very important factor for our indices, which means that it can be considered as a good predictor to explain composite accessibility and satisfaction related to healthcare. In this study, approximately 46 % of interviewees had health insurance. Out of the group with insurance, 60 % had public health insurance, 22 % had private insurance, and 18 % had both kinds of insurances.

## Discussion

The perceived time-decay parameter proposed in this study is a novel approach using qualitative individual data (perceived travel time) and quantitative data (gravity models-related data and spatial interaction data). We proposed to use the travel time to healthcare services reported by our interviewees. Few accessibility studies have used individual interview data in the construction of new spatial accessibility measures (Hawthorne and Kwan 2012), and the use of perceived measures may engage pluralist approaches to better understand healthcare accessibility from the patient´s perspective (Hawthorne and Kwan 2012).

The two indices obtained offer an integral perspective on health inequalities in the study area, and are constructed in a manner that allows the construction of similar indices for other cases studies. Furthermore, these indices can be enriched with more detailed information. Our indices are more sophisticated than simple indicators; they encompass indicators related to perceptions and behaviors, and their values represent complex phenomena of healthcare access and healthcare satisfaction.

In general, medium and low values of the CHCA and CHCS indices were found in southern areas, as well as in the extreme north-west of the city. The south of Quito city is an industrial–residential zone, inhabited by people with lower socio-economic conditions than the people living in the north (Cabrera-Barona et al. 2015; Lozano Castro 1991). The extreme north-west of the city is also characterized by socio-economically deprived neighborhoods. We interpret these results from a multidimensional perspective: our indices also respond to different social and demographic variables. More deprived neighborhoods tend to be related to low health outcomes (Collins et al. 2009; Wilson et al. 2004), whereby a health outcome is the satisfaction with the healthcare service.

The values of the CHCA index had high variations in the center of the city. This situation could be related not only to higher variations of index indicators, but also to the presence of an important number of healthcare services in this zone. Indeed, the center of Quito is the zone that has the highest concentration of public healthcare services in the city. Having more healthcare services in an area may foster, for example, higher variability of indicators of people´s acceptability related to these services. An area with the lowest values of the CHCA index was found in the south of Quito. The reason for this low healthcare accessibility could be the low density of health services in the extreme south of the city. Another reason could be the long appointment waiting time to receive healthcare that interviewees reported in this zone (less availability of the healthcare service). A third reason could be related to less confidence in the health services: most interviewees in this area reported that they decided to go to the chosen health services because the services were located very close to their households and not because they had confidence to those services.

In the case of the CHCS index, the differences between its different values are not pronounced. However, an area located in the north of the city had the lowest values of the CHCS index. It is important to note that this area does not necessarily represent an area where interviewees did not receive a good quality of care by physicians. It is possible that the waiting time to receive healthcare after arriving at the healthcare service influenced the index and reduced its values: in this zone all of the interviewees reported having received a good or excellent healthcare service by the physicians. Nevertheless, 73 % of the interviewees waited one hour or more at the healthcare service before receiving attention. Unacceptably long waiting times reduce the composite satisfaction with healthcare.

Our two indices can be considered as important tools for integral models of healthcare accessibility. Satisfaction with healthcare services and the characteristics of the health services have been used in analyses of spatial access to healthcare (Rosero-Bixby 2004). Access perceptions have also been found to be useful when evaluating healthcare accessibility (Comber et al. 2011; Hawthorne and Kwan 2012) due to the need of understanding different problems encountered by people in relation to healthcare access.

In developing our indices, we dealt with two complex healthcare processes: access and satisfaction. When using components of human spatial interaction and perceived travel time in the CHCA index, we expanded the traditional approach of calculating a distance-decay parameter to a more pluralistic approach that enriched the spatio-temporal and social analysis of healthcare accessibility. The CHCA index can be seen as an application of temporally integrated human geographies, a field with a big potential in supporting social scientists in performing different kinds of analyses (Kwan 2013). The use of perceived time also avoided data being missed in the households´ survey we carried out, because some interviewees had trouble recalling the distance they had travelled to the health service, and thus felt more comfortable only stating the time they travelled to the service. The CHCA index was able to capture interpersonal differences in accessibility. However, when calculating an accessibility index it is important to consider that different values of access depend strongly on the indicators used in the index, as well as on whether the analysis is based on the individual-level or not (Kwan 1998).

The perception of healthcare quality is influenced by emotional needs (Pascoe 1983), and complementing this perception with additional satisfaction-related information is important for gaining a better understanding of patient satisfaction. Even though there are studies that obtained information on healthcare satisfaction by conducting interviews inside health services (Elliott et al. 2012; Hekkert et al. 2009), our study obtained this information with interviews in households, due to the need for geo-referencing interviewees´ residences, in order to spatially relate the place where the interviewees live, the place where the health services are located, and the population (potential demand) of each of these places. Another advantage of this approach is that interviewees might be more comfortable answering the healthcare-related questions in their homes, rather than answering these kinds of questions at the health service.

In general, the results of our research show the presence of healthcare inequalities in the study area. Even though healthcare inequality is a general problem all over the world (Hare and Barcus, 2007; Carr-Hill 1990; Kakwani et al. 1997), it is important to mention that it is a very important issue in Latin America, due to the fact that Latin America has the highest disparities in terms of access to services, consumption levels, and other socioeconomic variables, of all regions in the world (Gasparini et al. 2011; Hoffman and Centeno 2003). Regarding healthcare inequalities, disparities in healthcare accessibility and healthcare satisfaction have been identified in other Latin American countries (Buzai 2011; Gómez 2002; Fuenzalida 2010; Rosero-Bixby 2004). Particularly in Ecuador, significant improvements have been made in the field of healthcare (Rasch and Bywater 2014). However, it is still very important to reduce socioeconomic inequalities, since these disparities exacerbate health inequalities, especially in marginalized communities in Ecuador (Parkes et al. 2009). These socioeconomic inequalities are also found in marginalized urban areas in important cities such as Quito (Cabrera-Barona et al. 2015).

We believe that the factors used to explain the indices developed in this investigation can lead to an improved evaluation of healthcare. The significant factors identified, such as age, marital status, education, and the need for care, belong to various socio-demographic dimensions, which confirms the importance of using a variety of social factors when explaining accessibility and satisfaction related to healthcare, even when some factors only offer a partial view of the social complexity related to healthcare.

Education, marital status and age are factors that have been proven to be associated with healthcare accessibility and satisfaction (Bleich et al. 2009; Bryant et al. 2002; Cavalieri 2013; Robinson and Thomson 2001). We found a positive and a negative relationship between the factors education (having a tertiary degree of education) and the marital status (being married), respectively, and the CHCA index. However, a previous study showed that single women are less likely to use a specific healthcare service (Bryant et al. 2002), while other studies did not find significant relationships between education and healthcare accessibility (Bryant et al. 2002; Cavalieri 2013). For these reasons, we can confirm that education and marital status are factors that are associated with the CHCA and CHCS indices, but final proof that these factors can have any positive or negative impacts on healthcare accessibility and satisfaction is still missing, and hence will be subject of future research.

The age and need factors are also associated with the developed indices, especially with the CHCS index. Age has been found to be associated with the patient´s expectations and the patient’s satisfaction (Bleich et al. 2009; Robinson and Thomson 2001). The need for healthcare is an ambiguous concept that varies between individuals and groups (Goddard and Smith 2001), and in the case of self-perceived health status, individuals that reported having poor health are less likely to be satisfied with healthcare (Bleich et al. 2009).

Having health insurance was found to be a very important factor for our indices, which means that it can be considered as a good predictor for explaining composite accessibility and satisfaction related to healthcare. In our study area, most of the interviewed people have public health insurance. Having public health insurance can enhance healthcare access by facilitating the access to healthcare services that belong to the Ecuadorian Social Insurance Institute. On the other hand, having private health insurance can be an option to supplement or complement public health insurance, especially in terms of long waiting lists in some public healthcare services (Cavalieri 2013).

Our results regarding health insurance are in agreement with other studies that identified health insurance tenure as an important factor for healthcare in Ecuador (De Paepe et al. 2012; López-Cevallos and Chi 2010; Waters 1999). Health insurance has been found to be a strong predictor of the use of healthcare services, such as preventive care services and hospitals (López-Cevallos and Chi 2010). It is important to point out that while the 2008 Ecuadorean Constitution (Constitution of Ecuador 2008) guaranteed access to healthcare for all citizens, universal health insurance is in reality still limited in some population groups (De Paepe et al. 2012). Our results, supported by previous research, show that health insurance coverage is a fundamental element in Ecuador to guarantee the use of healthcare services, particularly when there are still marked inequalities in terms of health. We also aim to show that our indices can identify healthcare inequalities considering a multidimensional perspective, and can interact with a variety of socioeconomic variables that may support healthcare planning.

## Conclusion

Multi-dimensional approaches in health planning are important due to the need for a better understanding of health inequalities. To the authors’ knowledge there is no prior research regarding accessibility and satisfaction related to healthcare that associates composite indices to different health behavior factors by applying different regression approaches in order to validate and explain these indices.

The indices generated in this study were useful to identify healthcare disparities in our study area. The differences in healthcare accessibility and healthcare satisfaction were analyzed and evaluated using social predictors and applying regressions. Regression results were consistent and strong: some social factors, mainly having health insurance, influence accessibility and satisfaction related to healthcare.

Before mentioning the significance of this study, it is important to mention some limitations we identified. We consider that our CHCA index can be improved by incorporating more detailed information of human transit between households and healthcare services in its accessibility indicator (*Acc*). We estimated human transit using population data and information regarding the position of households and healthcare services. However, human mobility cannot be fully understood by looking at where people live and where they can potentially go, and considering the complex mobility that a person can have during the day can be a useful source of information to evaluate healthcare accessibility. In the case of our CHCS index, we believe that more detailed information could be included in the healthcare quality indicator (*Q*), for example, information regarding healthcare staff qualities, such us listening skills, quality of interaction or helpfulness. All of these different characteristics could generate a more integral perspective of peoples’ satisfaction with healthcare.

It is also important to mention that we generalized the concept of healthcare service supply in our study to only consider the range of services. For example, a specialized hospital can obviously offer more services than a basic hospital. However, we aim that future CHCA and CHCS indices include the variable of healthcare service supply represented by different perspectives. For instance, for primary healthcare concerns, the healthcare service with the highest score could be a healthcare center and not a specialized hospital.

Our study can offer diverse contributions. The significance of this study lies not only in its contribution to the development of mixed-approaches that incorporate concepts and techniques from different fields, but it also offers a feasible operational method of calculating composite and multidimensional indices of healthcare accessibility and healthcare satisfaction. Another strength of this study is that the methodology could be applied in different contexts and situations, and is not limited to the Ecuadorean context. The information extracted from our survey and from the census data is information that may be available in other countries, especially in Latin American countries such as Argentina, Bolivia, Colombia, Chile, etc., since the production of Latin American census information shares basic conceptual aspects (CEPAL 1999; Martínez Pizarro and Calvelo 2012), and even when the census and survey information across Latin America is not entirely uniform, it can be adapted to allow a comparison between countries (Gasparini et al. 2011).

The indices developed in this study as well as our regression results lead to a better understanding of healthcare disparities or inequalities, and can thus be considered as important tools for decision makers towards more efficient decisions regarding the health system in the study area.

Future research related to these kinds of indices could include the incorporation of more detailed information regarding human mobility. It will also be important to relate these indices to information of socioeconomic deprivation in order to identify critical areas with high levels of deprivation and low levels of healthcare accessibility and satisfaction. Finally, another potential role of these indices could be the explanation of health inequalities in relation to specific health problems, such us chronic diseases or epidemics.
